# Developing and implementing a new methodology to test the affordability of currently popular weight loss diet meal plans and healthy eating principles

**DOI:** 10.1186/s12889-021-12447-4

**Published:** 2022-01-06

**Authors:** Ella L. Bracci, Rachel Milte, Jennifer B. Keogh, Karen J. Murphy

**Affiliations:** 1 Alliance for Research in Exercise, Nutrition and Activity, UniSA Clinical and Health Sciences, University of South Australia, Adelaide, SA 5001 Australia; 2 Caring Futures Institute, College of Nursing and Health Sciences, Flinders University, Adelaide, SA 5042 Australia

**Keywords:** Cost of diets, Affordability, Australia, Proportion of income, Food insecurity

## Abstract

**Background:**

Weight loss diets continue to rise in popularity; however, the associated costs are seldom reported. Certain weight loss diets may be unaffordable and differ from their traditional nutrition composition to include non-conventional premium products. In contrast, healthy eating principles such as the Australian Guide to Healthy Eating (AGHE) and the Mediterranean Diet (MedDiet) place an emphasis on fresh produce and staple foods but are sometimes thought to be unaffordable. A new methodology was piloted to assess the cost of weight loss diets using seven meal plans.

**Methods:**

Seven meal plans were analysed to quantify the absolute grams required of all ingredients across seven days and multiplied by the cost of the ingredient per gram to determine the total cost of each ingredient based on unit size and price. The weekly grocery shopping cost was determined through summation of all ingredients and their entire unit size to compare weekly costs.

**Results:**

Weekly meal plans (absolute grams) cost between $93-193AUD. The AGHE meal plan was the least expensive and 8 Weeks to Wow was the most expensive. Weekly grocery shopping of entire units cost between $345-$625AUD, over $100AUD greater than the spending of an average Australian ($237AUD/week).

**Conclusions:**

The financial feasibility for long-term sustainment of weight loss diets may be questionable for groups including low-income earners and low socioeconomic status. Further, when dietary patterns are adapted for weight loss, or followed by consumers, deviations from foundational principles tend to occur which may influence overall cost.

## Background

In 2017–2018, two thirds of the Australian adult population were overweight or obese and a higher proportion of adults were within the lowest socioeconomic areas [1, 2]. Overweight and obesity are risk factors for health complications but can be modified by nutrition and lifestyle [1, 2]. Resultingly there is demand for weight loss diets and meal plans to suit a variety of individuals. In 2011–2012, almost 2.5 million Australians reported having been on a weight loss diet, a higher proportion of which were females [3].

In recent times, nutrition and diet related advice has deviated from traditional and qualified health professionals such as nutritionists and dietitians towards social media and unqualified sources [4, 5]. It is therefore relatively unsurprising and concerning that the popular dietary advice from unqualified and underqualified individuals contradicts or deviates from the Australian government guidelines, the Australian Guide to Healthy Eating (AGHE) and the Australian Dietary Guidelines (ADG) [6–8]. However, it is important to acknowledge that qualified Australian health professionals may also provide dietary advice that differs from the national guidelines depending upon an individual’s medical history, food preferences, allergies and pre-existing intolerances.

The Australian government guidelines include a variety of staple foods such as breads, cereals, pasta, fruit, vegetables, legumes and dairy products that are generally priced affordably and are non-taxable (Government Services Tax-free) [9–12]. However, it is a common misconception that a healthy balanced diet, one balanced with core food groups and minimal discretionary foods is unaffordable and unobtainable, particularly depending on socioeconomic status (SES) and location [12]. But, research suggests that the prices of healthy foods in differing SES and income areas, may be equivalent to one another [13], or differ by up to 3% [9]. When Lee and Kane [9] compared healthy (modelled on the five AGHE food groups and foundation diet) with unhealthy (based on intake of Australians from the Australian Health Survey and inclusion of discretionary choices) diets in Queensland for five different household structures, the cost of the ‘healthy’ diet was more affordable for all households, likely due to the removal of alcoholic beverages, take-away foods and sugar-sweetened beverages. But, international data displays conflicting results as ‘healthy food’ in the UK has been reported to be consistently more expensive compared to less healthy foods, by up to £5 ($9AUD)[14]. In addition, Ni Mhurchu and Ogra [15] found that a healthy food basket based on core foods in New Zealand was slightly more expensive compared to the cost of a ‘regular’ shopping basket reflective of the population ($6AUD).

Contrastingly, research from Brazil has reported that minimally processed and unprocessed foods (Brazilian Real $4.28/kg) deemed to be healthy are more affordable when compared to processed (BR$7.64/kg) and ultra-processed foods (BR$6.92/kg) which may yield a less desirable nutrition profile [16]. Interestingly, some meal plans from popular diets once used for therapeutic purposes require the purchasing foods that are not standard practice, or outside of the norm. This includes low carbohydrate replacements including almond meal as opposed to regular wheat flour, or konjac (vegetable) noodles as opposed to egg or rice noodles, which can be significantly more expensive. Further, certain meal plans may encourage the purchasing of premium products such as ‘organic’ or gluten-free which significantly alter grocery shopping costs and wouldn’t typically be part of these diet. From the previous research, it is apparent that the cost of weight loss diets, meal replacements and weight loss programs can differ dramatically, with prices ranging from $70USD-$420USD/week [17] and up to $2100USD per quarter [18], and prices are affected by visits to health practitioners, memberships, duration of weight loss and levels of calorie restriction [18–20].

The evidence regarding the cost of a diet defined as heathy according to the ADG, versus a nonhealthy diet seems to be inconclusive and subjective. Previous research is limited, inconsistent and not standardised when calculating and reporting the costs of weight loss programs, dietary patterns and interventions. For example, research has identified the implementation of 12 different food pricing tools during 2003–2014 to calculate healthy food prices and affordability [21]. Further, differing protocols have been used, particularly regarding the modelling process and reference household structures on which the cost-effectiveness is based on [9, 21].

The present study aims to provide a standardised approach of costing seven popular weight loss diets based on meal plans for one adult. Diets will be compared based on their associated costs in relation to the available literature and similar studies. Further, we aim to assess the meal plans in comparison to Australian’s spending habits, government food basket initiatives and, briefly comment on food insecurity due to the current impact of covid19.

## Methods

### Identification and introduction of diets

To identify popular diets within Australia, Google Trend Data and grey literature were used during March to April of 2019. Specifications were used including search terms, date (2015–2019), web searches and geographical location (restricted to Australia) as previously published [22]. For similar diets i.e., Optifast and Optislim, the diet with the highest popularity (trending searches and relative search volume) from Google Trend, was selected. This tool has been previously utilised in research [23–28].

The meal plans in this research have previously been published and their nutrition profile reported [22]. The definitions and descriptions provided below are a combination of the diets scientific, or technical classifications, and their modern counterparts which may include foods that once were limited or excluded.

#### Popular weight loss diets

1. The Ketogenic diet is a typically a high fat, low to moderate protein, and very low carbohydrate diet. The dietary pattern limits glucose from carbohydrates to ensure that fat is the primary energy source, resulting in a metabolic state known as nutritional ketosis [29]. Fruit and vegetables, alongside breads, cereals, legumes, and grains are limited in a ketogenic diet to ensure total carbohydrate intake is typically < 50 g/day [30, 31]. The ketogenic diet has been endorsed by national [32] and international [33–37] bodies and has an established role in dietary management of chronic disease such as type two diabetes, cardiovascular disease, and epilepsy [31]. However, modern day ketogenic diets have become highly popularised and do not necessarily align with the true principles and nutritional guidelines. Modern day ketogenic diets are targeted for weight loss and encourage considerably higher protein intakes from animal products such as beef, chicken, turkey, and protein supplements [38]. Further, the sources of fat which forms most of a ketogenic diet are currently from popular higher cost products sources such as coconut oil, butter, cocoa butter, and ghee which have been popularised within mainstream social media [38].
2. A traditional Palaeolithic diet has no specific macronutrient recommendations. However, it has been hypothesised to be an approximate 27:31:45 macronutrient distribution of protein, carbohydrate, and fat respectively [39]. Due to the limiting of cereal products such as bread, pasta, and rice, for the purpose of the current research it will be referred to as a low carbohydrate diet. Previously known as the ‘caveman diet’, a traditional Palaeolithic diet limits processed foods and has a focus on eating fresh produce [40, 41]. This includes meat, fish, vegetables, seeds, nuts, and fruit. Some modern Palaeolithic diets and meal plans appear to include dairy products and legumes, while others do not. The palaeolithic diet has similarly been studied for its cardiometabolic role, weight loss efficacy, increased satiety and ability to beneficially modify the intestinal microbiome [40].
3. 8 Weeks to Wow (8WW) is a challenge style diet with an explicit food list that re-introduces restricted foods over a fortnightly period [18]. The dietary pattern is high in vegetables and protein with small amounts of dairy foods, fruit and breads and cereals. 8WW also includes various low carbohydrate alternatives and specialty products such as konjac (vegetable) rice and noodles, and, high protein bread (Protein Bread Co.)[18].
4. Intermittent fasting (IF) is also known as time-restricted feeding whereby there is an eating window followed by a period of fasting and abstaining from food. Reducing the eating window is believed to reduce energy intake (calories) due to the closeness of meals, and that fasting will induce a fat-burning state [19, 40]. Daily fasting windows may vary (16:8 or 20:4), or a varying approach may be utilised such as the 5:2 method [42]. The 5:2 method consists of two days of very low-calorie intake (500-600 kcal) followed by ad libitum consumption for the remaining five days. Generally, there are no food group restrictions.
5. Optifast is a meal replacement program designed for overweight and obese individuals and typically is a precursor for bariatric surgery [43, 44]. Despite this, Optifast products are available over the counter to the general population and are often utilised as a meal replacement weight loss strategy without the supervision of a dietitian or health professional. Optifast meal plans (< 800 kcal – 1500 kcal) can be described as moderate to high protein, moderate carbohydrate and low to moderate fat [43, 44]. Further, Optifast meal replacements are considered nutritionally complete, formulated to provide an array of essential vitamins and minerals and adequate protein to preserve lean muscle mass.

#### Healthy Eating Principles

1. The Australian Guide to Healthy Eating (AGHE) in conjunction with the Australian Dietary Guidelines (ADG) are evidence-based guidelines which promote a balanced lifestyle and reduce disease risk [45]. The five core food groups contribute to a moderate protein, moderate to high carbohydrate and moderate fat intake. No foods are restricted; however, it is recommended to reduce the intake of high fat, high sugar and high salt products and consume minimal amounts of heavily processed foods and alcohol.
2. The Mediterranean Diet (MedDiet) has been endorsed by the American Dietary Guidelines (2015–2020) as one of three dietary patterns to encourage healthy ageing and reduce disease risk [46, 47]. The MedDiet reflects a moderate protein, moderate carbohydrate, and high fat dietary pattern [20, 46]. A Mediterranean dietary pattern focuses on consuming ample amounts of olive oil, fruit, vegetables, cereals, wholegrains, legumes and smaller amounts of fish, eggs, and meat.

### Dietary Input

The full protocol has been previously published [22]. In brief, where various energy intakes (kJ/kcal) were available for diets, the most ‘suitable’ meal plan was selected based on the premise of weight loss (i.e., approximately 6090 kJ/1500 kcal). For example, the Optifast meal replacement program offers various ‘levels’ and energy targets. The 5040 kJ/1200 kcal meal plan was considered the most appropriate as 6300 kJ/1500 kcal is the ‘maintenance level’ and does not include any Optifast products. The 3360 kJ/800 kcal and 4200 kJ/1000 kcal meal plan were considered too drastic an energy deficit for an individual with a healthy body mass index (BMI) and therefore not selected.

Meal plans were entered into Foodworks Professional Dietary Software V9 (Xyris, Queensland, Australia) for analysis as previously described [22]. Where ingredients were missing or unavailable, the closest alternative was entered. The full dietary pattern for 8WW was entered to provide insight into the overall nutrition profile and cost. The AGHE did not have a specific meal plan but alternatively provided two exemplar days. The remaining five days were developed from available recipes provided by the Eat for Health website and the NHMRC.

### Cost methodology

To determine if there were any differences in food costs between two Australian leading supermarkets Coles and Woolworths, a random number generator was used to select *N* = 6 ingredients from each meal plan (*N* = 42 total). The same ingredients were costed for Coles and Woolworths and only recorded once to maintain consistency. The cost per gram between supermarkets was negligible (0.01 cents).

Foodworks data was exported into Microsoft Access. Ingredients were then sorted and collated prior to transferring into Microsoft Excel. While collating, the sum total of each ingredient that was used throughout the seven-day meal plan was calculated. Each diet was allocated a separate sheet where all ingredients were recorded once and listed alphabetically to ensure there were no duplicates. Ingredients from the excel spreadsheet were then cross-checked with the original exported list in Microsoft Access to ensure no ingredients were missing.

Details were then recorded from Coles Australia during July to August of 2019 including the unit size (mg, g, kg, mL, L), the cost of the unit ($) and the brand name. It was expected that all items noted in the meal plans would require purchasing including pantry staples such as salt, pepper, and cooking oils. Once the details of a single ingredient were obtained, it was incorporated into all meal plans to ensure consistency and accuracy as some ingredients can be purchased at various unit sizes. Further, to maintain consistency in the cost calculations, items on sale were avoided when possible and ‘middle’ range brands were used as opposed to premium or home-brand. However, in some circumstances, brand names may have been ‘temporarily unavailable’ therefore missing a price or were the same price as the home brand alternative. Furthermore, certain meal plans had non-specific ingredients such as ‘stock cubes’, therefore the average from multiple types of stock cube i.e. chicken, beef and vegetable were taken.

Average weights for fruits and vegetables that were not available pre-packaged with a designated weight-related price, were acquired from the Food Standards Australia New Zealand (FSANZ) food measures database [48]. The database provides approximate recorded weights of fresh produce that were used in the 2011–2013 Australian Health Survey and assisted in calculating the cost.

Once all ingredients had the required information in the excel spreadsheet, the cost per gram was calculated by dividing the cost by the unit size. For example, 400 g of almonds costs $10 and the cost per gram is $0.025. The cost per gram was calculated for all ingredients and then multiplied by the grams per week of the ingredient required by the meal plan from Foodworks Dietary Software program (Xyris, Australia) to calculate the cost per week. For example, 8WW requires 367 g of almonds over the entire meal plan ($0.025 × 367 g) with the cost per week totalling $9.20 for this specific ingredient. Once this was repeated for all ingredients, the sum was taken and resulted in the cost of the meal plan per week based on absolute grams.

Calculating the cost per meal plan based on absolute grams allowed standardisation amongst diets. But, as the meal plans require purchasing of entire unit sizes i.e. 400 g although only requiring 367 g, calculating the total cost of the grocery shop per week was integral as some ingredients would need purchasing of multiple units to meet the grams per week from the meal plan. This was evident in 8WW due to the increased frequency of meat consumption such as steak (fillet/blade) that was sold as $8/240 g and would require purchasing 8 units to meet the 1.8 kg requirement from the meal plan.

### Proportion (%) of income and contribution ($,%) from AGHE food groups

The average weekly income for an Australian adult before tax ($1659) as of February 2020 was applied to determine the percentage of average income required for diets [49]. A higher proportion of income was deemed less desirable as this indicates increased costs of a diet.

The final spreadsheet was used to determine the contribution in terms of cost ($) per food group and proportion of grocery shop (%) from the five AGHE food groups. Ingredients were allocated to one of the core five food groups, or, ‘other’ which represented the remaining ingredients which did not fit into the five food groups. The ‘other’ group included ingredients such as herbs and spices, condiments, oils, and alcoholic beverages. Once ingredients were listed under their respective food group, the cost was collated for ingredients i.e. the cost of apricots, apples, bananas, and all other sources of fruit, for each meal plan. To determine the percentage of cost for each food group, the total cost per food group was then divided by the total cost of the weekly shop. For example, across one week of the 8WW meal plan, fruit had an associated cost of $33 from the total $345 which equates to 9% of the total grocery shop.

Though tomato and avocados are classified botanically as a fruit, both are labelled as a vegetable according to the Western Australian Department of Health (Healthy WA) and are traditionally ‘used’ as a vegetable in main meals [50]. Further, the Queensland Healthy Food Access Basket (QLD HFAB) displayed both tomatoes and avocadoes within the vegetables food group [51] and was used to assess diets against reference costs.

Legumes and beans are categorised into both the vegetable food group and the lean meats and alternatives food group as per the AGHE. But, considering the QLD HFAB was used as a reference, legumes were considered part of the vegetable group for this particular calculation to ensure consistency [51].

In the circumstance that meal plans included foods that are non-traditional sources of an AGHE food group, for example, konjac noodles, or, protein pizza bases, and other pseudo-grains, these products were presented in the discretionary category.

## Results

### Cost of meal plans, grocery shop and proportion of income

The AGHE WL was the cheapest meal plan at $93 per week followed by ketogenic and the MedDiet WL at $112 per week. (Table 1) 8WW had the highest cost per week at $192, $100 more than the cheapest meal plan.

**Table 1:** Summary table of the cost of meal plans per week based on ingredients and absolute grams, the cost of the total food shop per week, and, cost of each diet expressed as a percentage of average income ($)

**Diet**	**Cost of Meal Plan Per Week ($/g)**	**Total cost of food shop per week ($)**	**(%) average income**^**b**^
Ketogenic	111.70	475.90	28.7
Palaeolithic	114.30	435.00	26.2
Intermittent Fasting	118.90	475.90	28.7
8 Weeks to Wow^a^	192.40	345.30	20.8
Optifast	125.10	624.90	37.7
MedDiet WL	112.00	452.00	27.2
AGHE WL	93.10	404.40	24.4

($/g) refers to the cost per gram of all ingredients in the meal plan while ($) cost of total food shop refers to purchasing of entire food or beverage unit

^a^Cost based on average of eight weeks for 8WW, individual weekly costs may differ

^b^Average income (before tax) based on 2019 ABS Australian data Published 20th February 2020 ($1659), *WL* weight loss, *AGHE* Australian guide to Healthy Eating

When looking at the total cost of food per week, that is purchasing the entire unit size of ingredients, not the exact gram amount, as per the supermarkets, the results differ. As food cannot be purchased in exact gram amounts needed for recipes and meal plans, it requires the purchasing of a whole packet, or ‘unit’ that the food manufacturer has established (i.e. a 400 g can of chickpeas or a 250 g bag of almond meal) which affects the total cost. The second cheapest grocery shop per week was 8WW ($345), however, this is the average over eight weeks and variation within weekly grocery shops will occur due to the reintroduction of foods. Contrastingly, the most expensive total cost was Optifast, costing > $600, followed by ketogenic and intermittent fasting ($476/week) (Table 1).

The proportion of income required for the total grocery shop ranged from 21% (8WW) to 38% (Optifast) with a higher proportion of income viewed as a negative outcome. Optifast was the only diet to surpass the 30% income threshold with the majority (4/7) of other diets falling between 25 to ≤ 30% income (Table 1).

### Highest contributions to cost per diet and non AGHE Food Groups

The most expensive ingredients for most diets were animal proteins such as chicken breast, salmon, steak, canned tuna and lamb. The ketogenic meal plan included a whey protein supplement ($23/400 g) and cocoa butter ($13/250 g) which were the highest contributors to cost for the ketogenic meal plan. Similarly, Optifast products are unable to be sourced from supermarkets, but can be found in Australian chemists and pharmacies, at a higher expense ($45-$65/636 g-954 g depending on the flavour and unit size). The purchasing of multiple different flavours within the meal plan provided an additional cost.

Interestingly, the three most expensive ingredients from the intermittent fasting meal plan were non-animal-based products, chia seeds (black and white) and coffee powder. The Intermittent Fasting meal plan had a significant amount of food and beverage products that did not fit into the AGHE categories ($233 of total food cost) (Table 2). The majority of these products and ingredients consisted of herbs such as mint, parsley, oregano, chilli, cinnamon, cloves, and, sauces including soy sauce, sriracha, tabasco sauce and Worcestershire sauce. Similarly, The ketogenic meal plan, ‘other’ category also had a high-cost contribution, requiring up to $289 of the total food cost per week (Table 2). This included a range of high fat dairy products (cocoa butter, double cream and ghee) and high salt products (prosciutto, bacon, pork rind). In a similar manner, the palaeolithic meal plan consisted of a range of high saturated fat coconut products such as coconut cream, coconut milk and coconut oil, but, did not include traditional dairy food sources. A range of dairy food alternatives such as almond milk and soy cheese were included as part of the meal plan, with an associated increased cost.

**Table 2:** Summary table of the cost ($) and percentage (%) contribution of total food shop ($) from meal plans by AGHE food group

		AGHE Food Group* $ Grocery Shop (% grocery shop)				
**Diet**	Fruit	Veg&L	B,C,G	LMP	DaA	Disc/Other ($)
QLD HFAB	16 (13)	34 (27)	16 (13)	39 (31)	18 (14)	73
Ketogenic	15 (8)	41 (22)	nil	97 (52)	33 (18)	289
Palaeolithic	17 (8)	72 (33)	nil	126 (59)	nil	220
Intermittent Fasting	32 (13)	82 (34)	26 (11)	62 (25)	42 (17)	233
8 Weeks to Wow*	33 (11)	72 (25)	10 (3)	141 (48)	37 (13)	53
Optifast	47 (21)	134 (60)	2 (1)	20 (9)	21 (9)	400
MedDiet WL	32 (11)	104 (36)	19 (7)	106 (37)	27 (9)	164
AGHE WL	26 (10)	102 (39)	35 (14)	58 (22)	38 (15)	145

^*^QLD HFAB Queensland Healthy Food Access Basket, 2014 ($126/week), costs are based on a one-person household with income $611.76 per fortnight (government assistance only)

*Veg&L* Vegetables and legumes, *B,C,G* breads, cereals, grains, *LMP* lean meats, poultry, seafood, legumes, *DaA* dairy and alternatives, *Disc/Other* Discretionary or other foods that do not fit into one of the five food groups *Percentages of AGHE Food Group does not include the cost of the discretionary/other category, only the AGHE food group costs, i.e. the total cost of fruit, vegetables, BCG, LMP and DaA

Though the 8WW diet similarly follows a low carbohydrate, high protein eating pattern there were minimal ingredients that did not fit into the AGHE food groups. Most products that were in the ‘other’ category consisted of low carbohydrate replacements such as konjac rice and Protein Bread Co products, and coconut oil.

The AGHE, MedDiet and Optifast meal plans had a cost associated with alcohol for cooking purposes and was within the topmost expensive products (white wine, brandy, and dry sherry).

### Cost of total grocery shop by AGHE food group

Fruit from meal plans contributed to 8–21% of the total weekly grocery shop or $15-$47 while vegetables contributed 22–60% ($41-$134) (Table 2). Breads, grains, and cereals had the lowest proportion of spending allocated ranging from nil to 14%, or $0-$35, while lean meats, poultry, and seafood, generally had the highest cost contribution ($20-$141) and proportion (9–59%).

The MedDiet WL, AGHE WL and Intermittent Fasting had similar proportions of cost attributed to each food group and were similar to that of the QLD HFAB (Table 2). The ketogenic and palaeolithic meal plans had similar cost proportions for fruit (8%), vegetables (22–33%), and the lean meats and poultry AGHE food group (52–59%). Further, both diets excluded breads, cereals and grains and therefore had no contribution to total cost. The palaeolithic meal plan additionally had no contribution from dairy and alternatives and differed substantially from the QLD HFAB.

8WW and Optifast required similar amounts for breads, grains, and cereals (1–3% or between $2–10), compared to the QLD HFAB (13% or $16), but differed noticeably in regard to lean meats and poultry (48% compared to 9%) and vegetables (60% compared to 25%) (Table 2).

## Discussion

In this study, the cost of the seven meal plans ranged from $93-193AUD (exact grams). The Australian Guide to Healthy Eating meal plan was the least expensive and 8 Weeks to Wow was the most expensive. Weekly grocery shopping of entire units cost between $345-$625AUD, over $100AUD which is substantially higher than the spending habits of an average Australian.

Countless meal plans for different weight loss diets exist due to consumer demand. However, not all meal plans are affordable, nor do they necessarily follow their original nutritional foundations. Namely, modern day ketogenic and palaeolithic weight loss meal plans appear to have a focus on high protein (from animal sources) and include a variety of low carbohydrate replacements and premium products which wouldn’t typically be included as part of either dietary pattern. Resultingly, these low carbohydrate meal plans may appear to be of a higher cost and unobtainable. Further, the Optifast meal plan utilised multiple different flavours of meal replacement which may be a useful strategy for diet adherence, but may be unaffordable, requiring a higher proportion of income. Additionally, the intermittent fasting meal plan which one would anticipate as cost-saving due to decreased food intake, included specialty and organic products, which add an additional expense and may not be necessary for weight loss. Access to information concerning the cost of meal plans and weight loss diets may assist in better informing consumers.

### Cost in relation to food security, income and AGHE good groups

Highly priced diets and meal plans may be financial barriers for some Australians. Food insecurity affects up to 5% of Australians at any given time and refers to an inadequate access to food that is affordable and culturally appropriate [52–54]. However, according to Foodbank, the largest food relief organisation in Australia, up to one fifth (21%) of the population may be experiencing food insecurity [55]. Food insecurity can be intensified during global pandemics such as COVID-19. According to the latest FoodBank report, the main groups that were accessing food relief have diversified with the increase of vulnerable Australian’s due to job cuts, businesses closing and lockdowns [56]. Previously, predominantly unemployed, homeless and single-parent families were accessing food relief; however, this has now expanded to include international students and casual workers [56].

For low-income earners, money dedicated to food shopping is not normally prioritised as housing expenses, fuel and power, health care, rent repayments and income tax are of a higher priority [9, 57]. Research suggests spending greater than 25% of disposable income on groceries and food supplies can lead to food insecurity [58–60]. However, a healthy diet can cost between 21–30% of disposable income for low-income families [9].

The Optifast meal plan required a significant proportion of income. However, previous research using partial meal replacements (Slim-Fast Foods Co) indicated that there may be suitability for lower income earners [61]. Further, previous research has indicated that weight loss diets and diets that follow a low-carbohydrate high-fat pattern are costly [62, 63]. One study reported the feasibility of a palaeolithic diet for low-income earners. The results found that a palaeolithic diet is of a higher cost ($0.23/100 g) compared to the observed diet of Americans ($0.17/100 g) [62], but, could still be feasible for low-income earners within the constraints of the thrifty food program, in this specific circumstance [62]. Further, Zinn et al. (2020) [63] compared the cost of a meal plan based on the ministry of health (MOH) nutrition guidelines and a LCHF diet similar to that of a palaeolithic or ketogenic diet. Comparable to that of the current research, the lack of traditional carbohydrate sources (grain foods) in low carbohydrate diets resulted in increased proportions of grocery shopping from other food groups, namely lean meats, poultry, and ‘other’. However, there are dietitian and medical professional led organisations such as the Noakes Foundation and Low Carb 4 Families which tailor culturally appropriate low carbohydrate dietary patterns for lower income population groups.

### Cost in relation to healthy food basket initiatives, and, home-brand compared to name-brand

In hopes to reduce the inequality of access to healthy diets according to SES, state, regional and community initiatives to improve access to healthy foods have been piloted and implemented [57, 60, 64, 65]. American initiatives such as the Supplemental Nutrition Assistance Program (SNAP), previously referred to as food stamps, and food baskets, provide assistance and access to nutritious foods for low SES families at a federal level [66]. Similarly, Australian government initiatives such as the Healthy Food Access Basket (HFAB) in Queensland (2014), demonstrate the ability and achievability of constructing a nutritionally adequate, low cost food basket that is in line with recommendations (AGHE)[51, 67]. Further, in Australia, a goods and services tax (GST) of approximately 10% is added to most non-core foods that are processed including pre-prepared foods, confectionary, savoury snacks, bakery products and sugar-sweetened beverages (SSB) [9, 59]. Most core and staple foods such as fruit, vegetables, bread, meat and poultry, and, dairy foods (milk and eggs) are non-taxable (GST-free) in an effort to avoid any GST related price rises on these foods [9–11]. However, this research demonstrates that certain diets and meal plans tend to require a lower proportion of weekly grocery costs from the five core food groups.

Affordability of diets and food is also influenced by purchasing tendencies [68]. Purchasing organic foods and premium products can increase costs dramatically [68] as noted in 8WW, which required purchasing of Protein Bread Co products, large amounts of chicken breast, lean beef and quark (a dairy product comparable to cream cheese). Similarly, the Optifast meal plan recommended a variety of meal replacement flavours requiring the purchase of multiple boxes ($45-$60 per flavour depending on the unit size). In addition, the ketogenic meal plan included two different flavours of premium brand whey protein isolate (WPI) which wouldn’t typically be part of a ketogenic diet. The remaining diets also had premium and or unusual products which were difficult to source a price from Coles, Australia. This included soy cheese (palaeolithic), cocoa butter (ketogenic), liquid smoke sauce (ketogenic), bliss balls (Intermittent Fasting), organic corn chips (Intermittent Fasting) and vine leaves (MedDiet).

In contrast, purchasing generic brands and non-premium products can decrease costs by up to 13% ($23-$32) per week [65]. When the Queensland HFAB was adjusted to include generic brands, the cost of the basket reduced by 18% ($11.40), resulting in an annual saving of $594 [51, 67]. For example, almonds, a common food product in all meal plans differs substantially in price depending on Coles brand ($17.50/1 kg) or name-brand ($22.00/1 kg). Similarly, per kilogram of chicken breast, choosing home brand ($12.00/kg) can result in a saving of almost $20 compared to the premium equivalent ($31.50/kg). In addition, frozen goods and bulk buying can reduce costs, and may result in increased affordability of the researched diets [69]. However, although a cost saving idea in theory, bulk-buying may only be appropriate for non-perishable (staple items) such as grains, legumes, rice, pasta, and canned goods, and requires adequate space for storage. Staple items are often excluded from popular weight loss diet trends such as a modernised Ketogenic and Palaeolithic diet and 8WW and therefore this strategy may not influence food costs.

Access to information regarding the costs of certain diets and the proportion of income required to undertake such dietary patterns could be a useful tool to inform health professionals and consumers. This information may assist in decision making and provide a deeper understanding of the financial costs of currently popular diets in Australia, addressing a gap in the literature. Further, this information could help to determine the suitability for certain groups and populations such as low-income earners, students, retirees and health-conscious consumers and the relevant risk for food insecurity.

## Limitations and conclusions

Previous research has estimated the cost of dietary patterns and meal plans, weight loss medications (Orlistat), weight loss programs (Jenny Craig, Weight Watchers) and meal delivery services (Lite N Easy, DineWise ©, Diet to Go©, Chef’s Diet™) [17, 70–73]. However, methodologies and cost calculations differed as some used mathematical optimisation models, others were based solely on the cost of meal plans and delivery fees, or medications over a certain timepoint [17, 62, 70–73]. Further, the units differed between research articles. Units and reporting of cost results ranged from timepoint i.e. per three months, five days, twenty-eight days, caloric level i.e. 1200 kcal compared to 1800 kcal, cost per kilogram of weight lost, cost per meal, and cost per week based on the quantity of sessions with a health professional required to achieve the desired weight loss [17, 71, 73, 74]. It was therefore difficult in some circumstances to compare the results of previous research to the current research.

The definition and cost of a healthy diet is relatively inconsistent in the literature. But, the healthy food baskets and government assistance programs from various governments can act as a reference point and cost comparator for weight loss diets due to the inclusion of core foods that align with dietary guidelines. Though some meal plans such as 8WW and ketogenic required premium specialty products and are more expensive than healthy eating principles by up to almost AUD$100, they may not put Australians at the risk of food security as they are below the 30% of income threshold. However, individuals have different priorities regarding disposable income, and, the cost required to undertake a certain dietary pattern may be appealing based on these results, or, a concern and viewed as a financial barrier.

## Data Availability

The datasets generated and analysed during the current study are available from the corresponding author on request.

## Abbreviations

AGHE: Australian Guide to Healthy Eating
MedDiet: Mediterranean Diet
AUD: Australian Dollars
ADG: Australian Dietary Guidelines
SES: Socioeconomic status
BR: Brazillian Real
USD: United States Dollars
8WW: 8 Weeks to Wow
IF: Intermittent Fasting
Kcal: Kilocalories
kJ: Kilojoules
BMI: Body Mass Index
FSANZ: Food Standards Australian New Zealand
HFAB: Healthy Food Access Basket
WL: Weight Loss
ABS: Australian Bureau of Statistics
NZ: New Zealand
HCLF: High Carbohydrate Low Fat
MOH: Ministry of Health
SEIFA: Socioeconomic Index for Areas
